# A rare case of esophageal metastasis from signet-ring cell carcinoma of the cecum

**DOI:** 10.1186/s40792-023-01768-8

**Published:** 2023-10-24

**Authors:** Yoichi Tanaka, Osamu Chino, Hiroshi Kajiwara, Tomoko Hanashi, Tomoki Nakamura, Hiroyasu Makuuchi

**Affiliations:** 1 Department of Surgery, Tokai University Tokyo Hospital, 1-2-5 Yoyogi, Shibuya-ku, Tokyo, 151-0053 Japan; 2 Department of Pathology, Tokai University School of Medicine, 143 Shimokasuya, Isehara, Kanagawa 259-1193 Japan; 3 Tokai University School of Medicine, 143 Shimokasuya, Isehara, Kanagawa 259-1193 Japan

**Keywords:** Metastatic esophageal cancer, Colorectal cancer, Signet-ring cell carcinoma, Submucosal tumor, Intramural metastasis, Immunohistochemistry

## Abstract

**Background:**

Metastatic esophageal cancer is rare. Its common primary lesions include lung cancer and breast cancer. Metastatic esophageal cancer originating from colorectal cancer is rarer.

**Case presentation:**

A 79-year-old woman visited our hospital because of lower abdominal discomfort. She was endoscopically diagnosed with type 0–IIa + IIc cancer of the cecum, and biopsy of the lesion showed signet-ring cell carcinoma. With a preoperative clinical staging of cStage I (cT2, cN0, cM0), the patient underwent laparoscopic ileocecal resection with D3 lymphadenectomy. Histopathological examination of the resected specimens revealed signet-ring cell carcinoma [type 4, pT4a, pN3 (No. 203), M0, pRM1, stage IIIc, R1]. Despite radial margin positivity, the patient refused resection of the residual tumor and received oral tegafur and uracil. *KRAS* mutation test showed *KRAS* wild-type colon cancer, but she refused anti-epidermal growth factor receptor therapy. One year after surgery, her blood carcinoembryonic antigen concentration elevated. Colonoscopy showed anastomotic recurrence and biopsy of the lesion showed signet-ring cell carcinoma. Upper gastrointestinal endoscopy showed multiple longitudinal submucosal tumors with erosions on their surfaces in the esophagus. Tumor biopsy revealed signet-ring cell carcinoma. Immunohistochemistry showed that the histological type of the esophageal tumors was the same as that of the primary colon cancer. Based on these findings, the esophageal tumors were diagnosed with metastasis from signet-ring cell carcinoma of the cecum. The oral chemotherapy was replaced with FOLFOX plus bevacizumab. However, the patient’s condition required treatment discontinuation, and she died of cancer progression 1 year and 5 months after surgery.

**Conclusions:**

To our knowledge, this is the first case report on metastatic esophageal cancer from signet-ring cell carcinoma of the cecum. Esophagoscopy showed multiple longitudinal submucosal tumors, which is similar to an endoscopic finding of intramural metastasis from primary esophageal cancer. We consider that the multiple longitudinal submucosal tumors are a notable feature of our case. When metastatic esophageal cancer is suspected, clinicians, endoscopists, and pathologists should consider signet-ring cell carcinoma of the colon as one of potential primary lesions. This consideration could lead the specialists to appropriate examinations and treatments, thereby improving clinical outcomes in patients with the metastasis.

## Background

Metastatic esophageal cancer is one of uncommon cancers. Mizobuchi et al. reported in 1997 that esophageal metastasis was found in 6.1% of Japanese autopsied patients who died of cancer [1]. In their cases, the most common primary lesion was lung cancer, followed by breast cancer and gastric cancer [1]. The researchers found that esophagoscopy was helpful for making a diagnosis of metastatic esophageal cancer and that esophageal stenosis with intact mucosa was endoscopically characteristic of the cancer [1].

We experienced a rare case of metastatic esophageal cancer originating from signet-ring cell carcinoma of the colon. To the best of our knowledge, this is the first case report on the metastatic cancer in the English language. In this paper, we report our case and compare it with previously published cases of esophageal metastasis from colorectal cancer. We also report multiple longitudinal submucosal tumors of the esophagus, which was a notable endoscopic feature of our case, with some literature review.

## Case presentation

A 79-year-old Japanese woman visited our hospital because of lower abdominal discomfort. She was endoscopically diagnosed with type 0–IIa + IIc cancer of the cecum, and biopsy of the lesion showed signet-ring cell carcinoma. Abdominal computed tomography showed no serous involvement, peritoneal dissemination, abdominal lymph node metastasis, or distant metastasis from the colon cancer. Based on all these findings, we determined that the preoperative clinical staging of the cancer was cStage I (cT2, cN0, cM0).

**Fig. 1:**
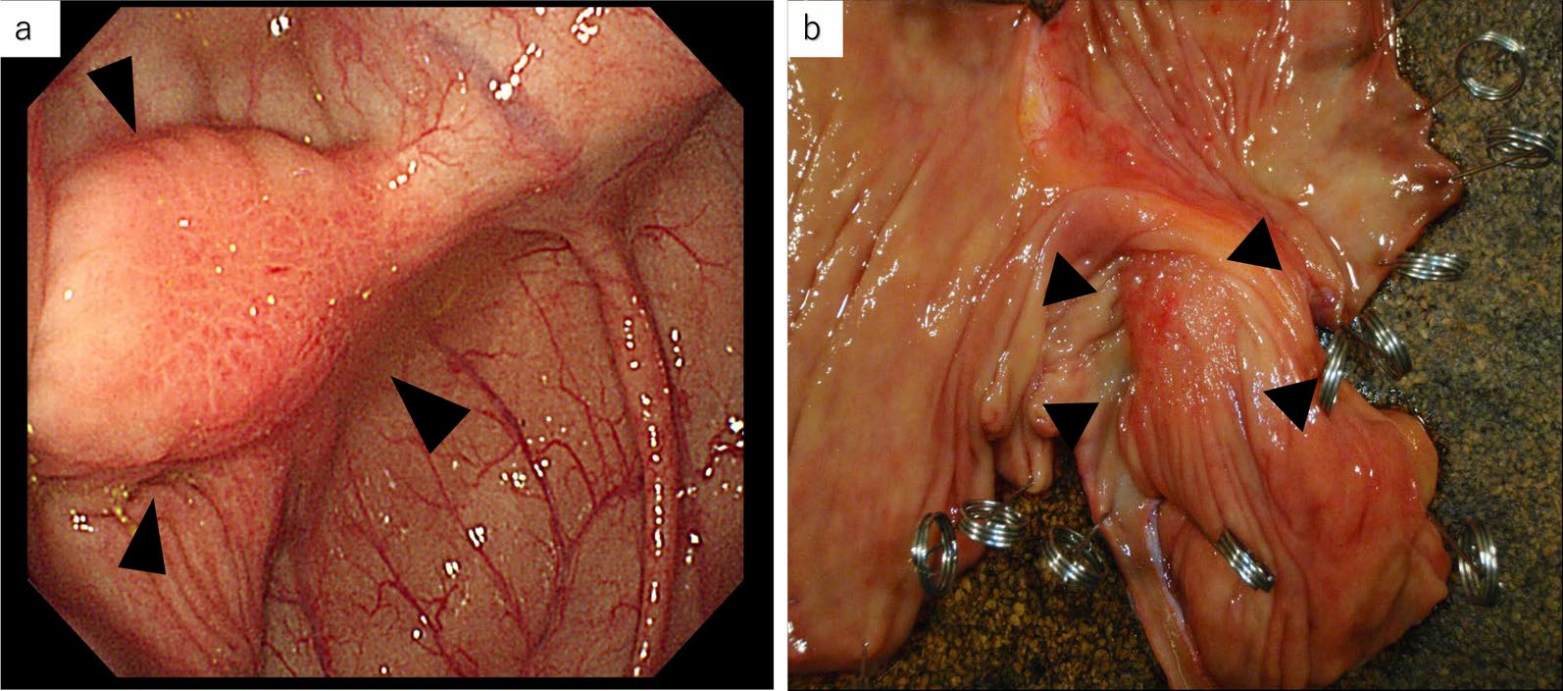
Macroscopic findings of the colon tumor. **a** Conventional endoscopic view of the colon tumor at the initial examination. **b** Macroscopic view of the resected specimen of the colon tumor. Both views show a 25-mm type 0–IIa + IIc tumor near the appendiceal orifice in the cecum (arrowheads)

The patient underwent laparoscopic ileocecal resection with D3 lymphadenectomy (Fig. 1). No residual tumors were macroscopically found in the radial margin during the surgery. Histopathological examination of the resected specimens showed signet-ring cell carcinoma [type 4, pT4a, pN3 (No. 203), M0, pRM1, stage IIIc, R1] with severe lymphatic involvement, many lymph node metastases, and radial margin positivity. We suggested resection of the residual tumor to the patient, but she refused it.

*KRAS* mutation test of the colon cancer revealed that the patient had wild-type *KRAS*. This result indicated that she was a candidate for anti-epidermal growth factor receptor (EGFR) therapy. However, she refused the therapy because of its potential adverse effects, such as acne-like rash. She also refused vascular endothelial growth factor (VEGF) inhibitors. She chose and began to receive oral chemotherapy using tegafur and uracil.

**Fig. 2:**
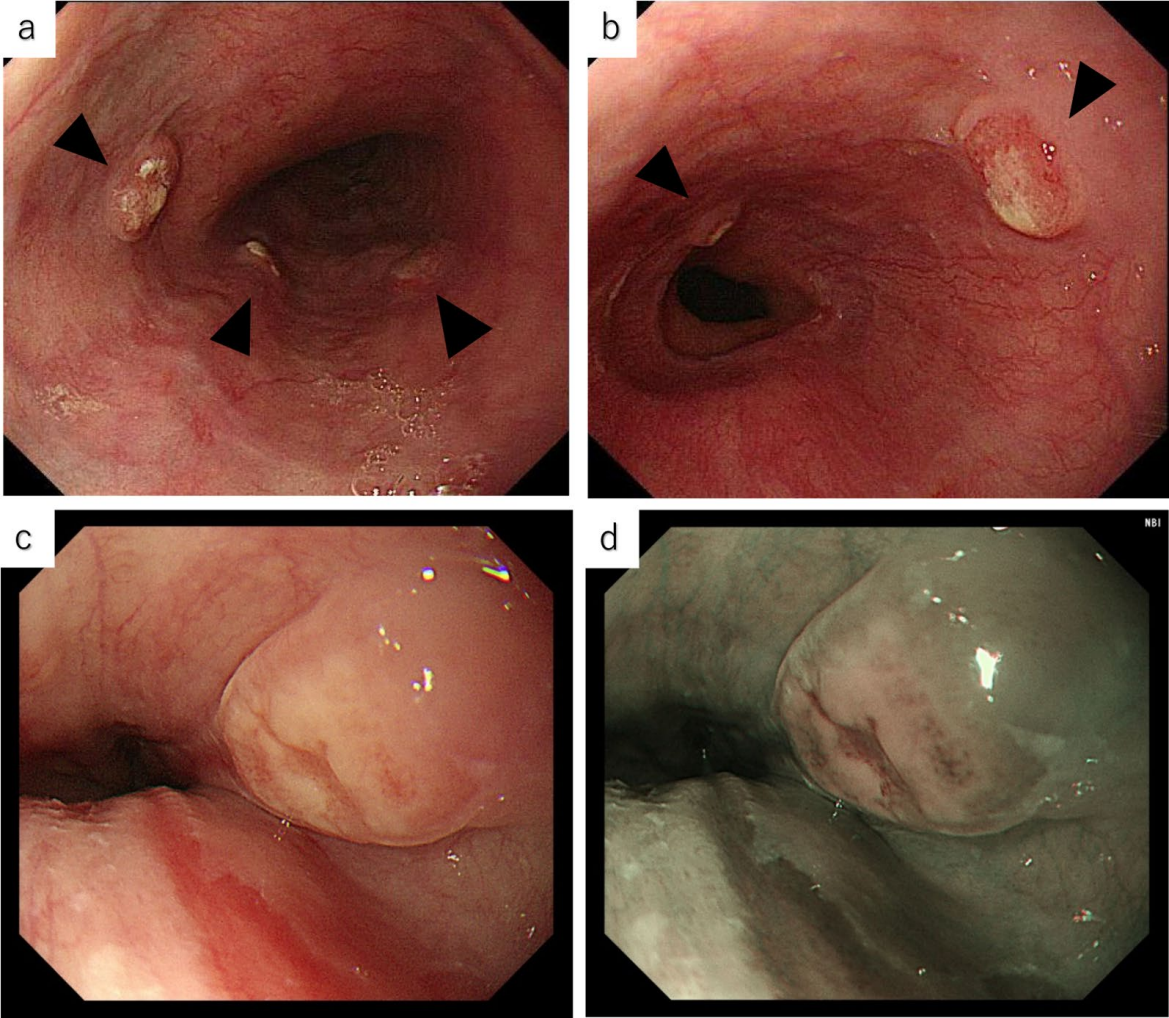
Upper gastrointestinal endoscopic findings. **a,** **b** Conventional endoscopic views showing multiple longitudinal submucosal tumors with erosions on their surfaces in the middle to lower esophagus (arrowheads). **c** Conventional endoscopic view of an esophageal tumor. **d** Narrow-band imaging endoscopic view of the esophageal tumor shown in Panel **c**

One year after surgery, the patient lost her appetite, and the blood carcinoembryonic antigen concentration elevated to 23.8 ng/mL. Colonoscopy revealed anastomotic recurrence, and biopsy of the lesion showed signet-ring cell carcinoma. Conventional upper gastrointestinal endoscopy with white light showed multiple longitudinal submucosal tumors with erosions on their surfaces in the middle to lower esophagus (Fig. 2a–c). Narrow-band imaging endoscopy of the esophagus showed that the elevated margins of the tumors were covered with non-neoplastic epithelia (Fig. 2d).

**Fig. 3:**
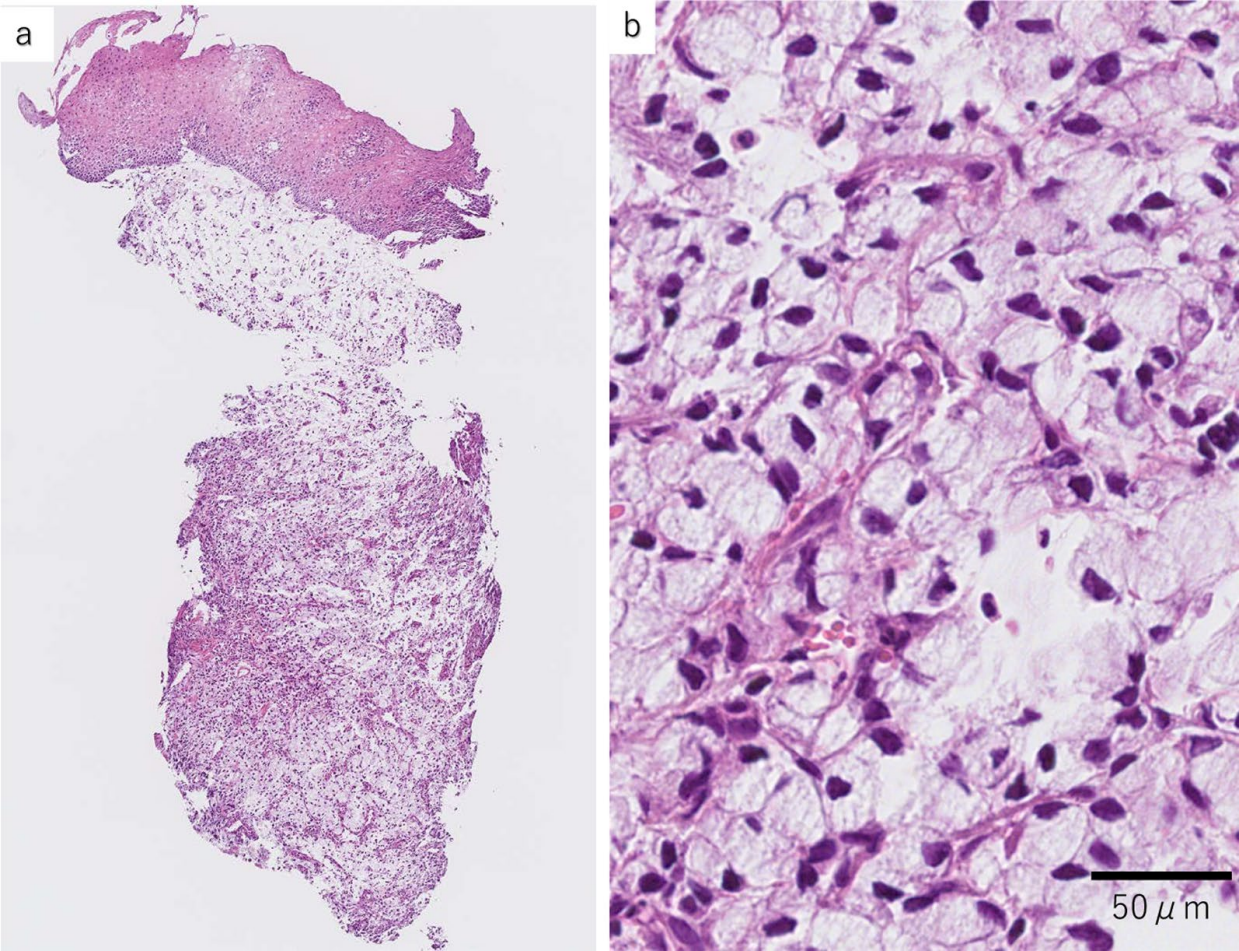
Hematoxylin–eosin staining results of biopsy specimens of esophageal tumors. **a** Low magnification view showing that the tumor is located predominantly in the submucosal layer and is continuous with the esophageal epithelium. **b** High magnification view showing signet-ring cell carcinoma

Biopsy of the esophageal tumors histologically showed that they were located predominantly in the submucosal layer, were continuous with the esophageal epithelium, and contained signet-ring cell carcinoma (Fig. 3). The histological type of the esophageal tumors was the same as that of the resected ileocecal specimens. Based on all these findings, we diagnosed the esophageal tumors with metastatic esophageal cancer originating from signet-ring cell carcinoma of the cecum.

**Fig. 4:**
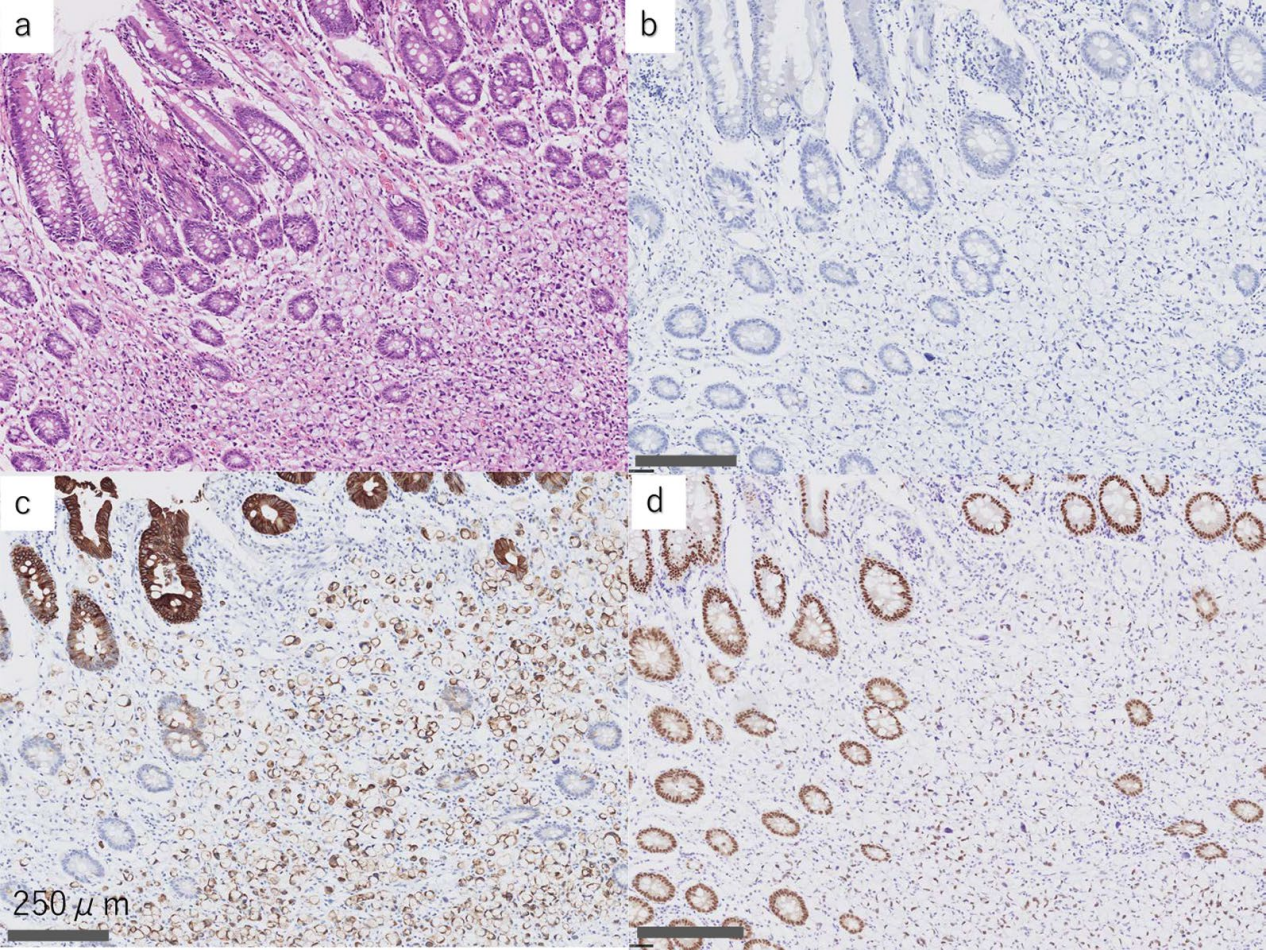
Hematoxylin–eosin and immunohistochemical staining results (high magnification images) of the resected specimen of the colon tumor. **a** Hematoxylin–eosin staining. **b** Negative cytokeratin 7 (CK7) staining. **c** Positive cytokeratin 20 (CK20) staining. **d** Positive caudal-type homeobox 2 (CDX2) staining. This CK7−/CK20 + /CDX2 + staining pattern is consistent with the staining pattern of signet-ring cell carcinoma of the colon

**Fig. 5:**
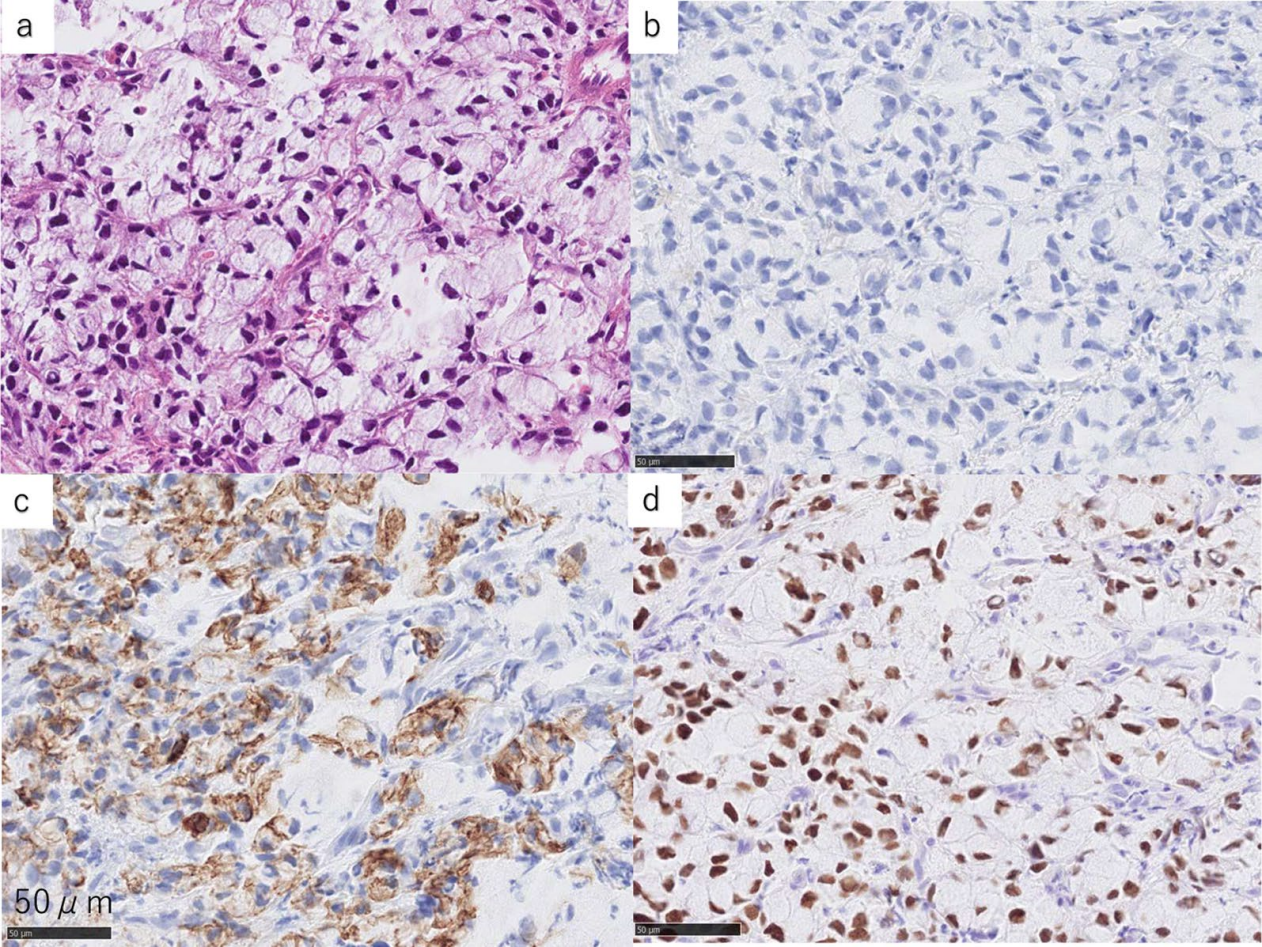
Hematoxylin–eosin and immunohistochemical staining results (high magnification images) of biopsy specimens of esophageal tumors. **a** Hematoxylin–eosin staining. **b** Negative cytokeratin 7 (CK7) staining. **c** Positive cytokeratin 20 (CK20) staining. **d** Positive caudal-type homeobox 2 (CDX2) staining. This CK7−/CK20 + /CDX2 + staining pattern is consistent with the staining pattern of signet-ring cell carcinoma of the colon

Immunohistochemical examination showed that both the primary cancer and its metastatic lesions were negative for cytokeratin (CK) 7 and were positive for CK20 and caudal-type homeobox (CDX) 2 (Figs. 4 and 5). These results correspond to the staining pattern of colorectal signet-ring cell carcinoma. Therefore, we diagnosed the patient’s condition with anastomotic recurrence and metastatic esophageal cancer after the resection of colon signet-ring cell carcinoma.

The patient did not respond to 1-year oral chemotherapy using tegafur and uracil, resulting in anastomotic recurrence and esophageal metastasis of colon cancer. She hoped to receive a VEGF inhibitor, and her chemotherapy regimen was replaced with FOLFOX plus bevacizumab (FOLFOX/BV). Subsequently, she broke her femoral neck in a fall, and underwent bipolar hip hemiarthroplasty. She started physical therapy, but her performance status declined. After 2 cycles of FOLFOX/BV therapy was finished, her chemotherapy was discontinued. Two months later (i.e., 1 year and 5 months after ileocecal resection), she died of cancer progression.

## Discussion

We report a rare case of metastatic esophageal cancer originating from signet-ring cell carcinoma of the cecum. We discuss characteristics of esophageal metastasis from colorectal cancer by comparing our case with previously published cases.

Metastatic esophageal cancer was first reported in 1942 by Gross and Freedman [2]. Subsequently, many cases of the cancer have been reported. A common first symptom of esophageal metastasis is dysphagia. Mizobuchi et al. reported that esophageal stenosis covered with intact mucosa was endoscopically characteristic of metastatic esophageal cancer [1]. Mechanisms of esophageal metastasis include lymphatic or hematogenous spread of the primary cancer in a distant organ. Metastatic esophageal cancer is often located in the submucosal layer covered with intact mucosa, which makes the diagnosis of the cancer difficult [3]. Differential diagnoses of metastatic esophageal cancer include esophageal submucosal tumors and rare types of primary esophageal cancer (e.g., basaloid squamous cell carcinoma, sarcoma, lymphoma). Ultrasonography has been reported to be useful for making a diagnosis of metastatic esophageal cancer [4]. The cancer is commonly treated with standard chemotherapy for the primary site. Esophageal stent placement is a treatment option for patients with malignant esophageal stenosis. However, the outcome of treatment is poor in most cases of esophageal metastasis.

In our case, the patient experienced no symptoms of esophageal stenosis, such as vomiting, dysphagia, and difficulties in eating. Although esophageal stenosis covered with intact mucosa has been known as a characteristic endoscopic finding of metastatic esophageal cancer, no such stenosis was found in our patient. She had multiple longitudinal submucosal tumors with erosions on their surfaces and with non-neoplastic epithelia on their edges. This finding is different from typical endoscopic findings of primary esophageal cancer or esophageal submucosal tumors, which usually occur as a single tumor.

Multiple longitudinal tumors of the esophagus have been found in patients with intramural metastasis from primary esophageal cancer. Watson et al. referred to longitudinal intramural metastasis from primary esophageal cancer [5]. Intramural metastasis of the esophagus is defined as “metastatic lesions in the esophageal, pharyngeal, or gastric wall macroscopically (clearly) separate from the primary tumor” in the Japanese Classification of Esophageal Cancer 11th Edition proposed by the Japanese Esophageal Society [6]. In our case, esophagoscopy showed that multiple submucosal tumors with mucosal changes extended longitudinally and discontinuously. This finding is similar to a common endoscopic finding of intramural metastasis from primary esophageal cancer. We consider that the multiple longitudinal submucosal tumors with mucosal changes are a notable feature of our case.

According to Takubo et al. [7], frequent lymph node metastasis in patients with intramural metastasis from primary esophageal cancer suggests that intramural metastasis is a secondary lesion of the affected lymph nodes as well as the primary cancer. In our case, many regional lymph node metastases and severe lymphatic invasion were found in the resected colon specimens. Signet-ring cell carcinoma of the colon has been reported to tend to severely invade lymphatic vessels in the abdomen [8]. We thus consider that our patient’s colon signet-ring cell carcinoma metastasized into the esophageal wall via the abdominal lymph flow from lymphatic vessels invaded severely by the colon cancer. The intramural metastasis could have appeared as multiple longitudinal submucosal tumors of the esophagus.

Metastatic esophageal cancer is typically covered with intact mucosa, which often makes differential diagnosis difficult. In our case, however, no magnifying endoscopy or ultrasonography was performed on the esophagus. Tumor biopsy enabled us to diagnose the esophageal condition.

In patients with esophageal metastasis from the gastrointestinal tract, the most common primary lesion is the stomach [1]. Esophageal metastasis from colorectal cancer is rare. A PubMed search of case reports found only 6 patients with such metastasis between 1976 and 2021 [9–14] (Table 1). The pathological diagnosis of primary colorectal cancer was compared in the 6 patients; 4 had moderately or poorly differentiated adenocarcinoma, 1 had mucinous adenocarcinoma, and 1 had no available data. None of the 5 patients with pathological data had primary signet-ring cell carcinoma of the colon. To the best of our knowledge, the present report documents the first case of metastatic esophageal cancer originating from signet-ring cell carcinoma of the colon in the English language.

**Table 1:** Cases of esophageal metastasis from colorectal cancer identified on literature review

Source	Age (year)	Sex	Primary site	Pathological diagnosis of primary site	Stage of colorectal cancer	Abdominal lymph node metastasis	Endoscopic finding of esophageal metastasis	Esophageal stenosis	Dysphagia	Treatment of esophageal metastasis
Fisher [9]	17	M	Rectum	PDA	IV	No data	Mild inflammation	Yes	Yes	Supportive care
Lohsiriwat et al. [10]	44	M	Rectum	MA	IV	Yes	Sessile polyp	None	None	Supportive care
Kagaya et al. [11]	55	M	Cecum	MDA	IV	Yes	SMT covered with normal mucosa	Yes	Yes	Esophageal stenting followed by esophagectomy with chemotherapy
Thomasset et al. [12]	62	M	Sigmoid colon	MDA	III	Yes	Soft tissue mass	None	None	Chemotherapy
Vashi et al. [13]	44	M	Sigmoid colon	No data	III	Yes	Multiple nodules	None	Yes	Chemotherapy
Watanabe et al. [14]	54	F	Rectum	MDA	III	Yes	SMT with white speckles	None	Yes	Chemotherapy
Present case	74	F	Cecum	SRCC	III	Yes	Multiple longitudinal SMTs with erosions	None	None	Chemotherapy

*MA* mucinous adenocarcinoma, *MDA* moderately differentiated adenocarcinoma, *PDA* poorly differentiated adenocarcinoma, *SMT* submucosal tumor, *SRCC* signet-ring cell carcinoma

Of the 6 patients, 4 had mucosal changes of the esophagus found by endoscopy. Two patients had esophageal stenosis. These results suggest that esophageal metastasis from colorectal cancer is likely to cause mucosal changes rather than stenosis of the esophagus. None of the 6 patients had multiple longitudinal tumors of the esophagus, which differs from our case. Five patients had lymph node metastasis in the abdomen. This finding confirms that the lymphatic spread is a common route for esophageal metastasis from colorectal cancer.

Primary signet-ring cell carcinoma of the colon is as rare as 0.1–2.4% of all colorectal cancers and has a poor prognosis [15]. In contrast, primary signet-ring cell carcinoma of the stomach is common, including minute signet-ring cell carcinoma [16]. For the identification of the primary site of metastatic cancer, immunohistochemistry using CD7, CD20, and CDX2 has become a useful examination [17, 18]. In our case, immunohistochemical examination was performed to differentiate colorectal signet-ring cell carcinoma from gastric signet-ring cell carcinoma as the primary site, although upper gastrointestinal endoscopy showed no notable abnormality in the stomach. Both the colorectal lesion and the esophageal tumors showed the CK7−/CK20 + /CDX2 + staining pattern, which suggests that the esophageal tumors originated from colorectal cancer [17, 18]. We found that immunohistochemistry using CD7, CD20, and CDX2 helpful for differentiating colon signet-ring cell carcinoma from gastric signet-ring cell carcinoma to identify the primary site of metastatic esophageal cancer.

Since the absence of esophageal stenosis and the results of immunohistochemical examination suggested that our patient’s metastatic esophageal cancer originated from signet-ring cell carcinoma of the colon, the esophageal lesion was treated with systemic chemotherapy and molecular targeted chemotherapy for the treatment of unresectable advanced or recurrent colorectal cancer in compliance with the Japanese treatment guidelines for colorectal cancer [19].

However, there were 2 major limitations of her treatment. First, anti-EGFR therapy could not be administered. The resected specimen of the colon cancer had wild-type *KRAS* mutation, indicating that the patient was a candidate for anti-EGFR therapy. However, she refused the therapy because of its potential adverse effects, such as acne-like rash. In addition, her primary tumor was right-sided colon cancer. Anti-EGFR therapy has been known to be less effective for right-sided colon cancer than for left-sided colon cancer [20]. Therefore, she was treated with FOLFOX/BV therapy. Second, neither *BRAF* mutation test nor *MSI* mutation test could be performed to identify effective treatment regimens for colon cancer. Both gene panel tests were not yet covered by the national health insurance and thus were not clinically available when we treated the patient.

## Conclusions

To the best of our knowledge, this is the first report on metastatic esophageal cancer originating from signet-ring cell carcinoma of the colon in the English language. Upper gastrointestinal endoscopy showed multiple longitudinal submucosal tumors of the esophagus, which is similar to an endoscopic finding of intramural metastasis from primary esophageal cancer. We consider that the multiple longitudinal submucosal tumors are a notable feature of our case. When metastatic esophageal cancer is suspected, clinicians, endoscopists, and pathologists should consider signet-ring cell carcinoma of the colon as one of potential primary lesions. This consideration could lead the specialists to appropriate examinations and treatments, thereby improving clinical outcomes in patients with the metastasis.

## Notice of previous case presentation

The same case was briefly presented in a review article of Japanese medical magazine in the Japanese language [21]. No text or figure of the article was reproduced in the present case report.

## Data Availability

Data that support the findings of the present study are available from the corresponding author upon reasonable request.

## Abbreviations

BV: Bevacizumab
CK: Cytokeratin
CDX: Caudal-type homeobox
EGFR: Epidermal growth factor receptor
FOLFOX: Folinic acid + fluorouracil + oxaliplatin
VEGF: Vascular endothelial growth factor
